# Pichia-CLM: A language model–based codon optimization pipeline for *Komagataella phaffii*

**DOI:** 10.1073/pnas.2522052123

**Published:** 2026-02-17

**Authors:** Harini Narayanan, J. Christopher Love

**Affiliations:** ^a^Koch Institute for Integrative Cancer Research, Massachusetts Institute of Technology, Cambridge, MA 02139; ^b^Department of Chemical Engineering, Massachusetts Institute of Technology, Cambridge, MA 02139

**Keywords:** recombinant protein production, codon usage bias, encoder–decoder networks, biotechnology, genetic sequences

## Abstract

The preference in synonymous codon usage—the so-called codon usage bias (CUB)—is governed by several factors such as the host organism, context and function of the gene, and the position of the codon within the gene itself. We demonstrated that this mapping can be learned from the host’s genome using language models and subsequently applied for codon optimization of heterologous proteins expressed by the host. This pipeline called Pichia–Codon language model (Pichia-CLM) was applied to the industrial host organism, Komagataella phaffii. With this approach, production of heterologous proteins was enhanced up to threefold compared to their native sequences. Furthermore, Pichia-CLM consistently yielded constructs with enhanced productivity for proteins of varied complexity, compared to commercially available tools. Finally, we showed that Pichia-CLM generates sequences resembling the properties of codon usage found in the host’s intrinsic host cell proteins and learned features such as avoiding negative cis-regulatory and repeat elements based on patterns in the genome data. These results show the potential of language models to unbiasedly learn patterns and design robust sequences for improved protein production.

## Significance

This paper presents Pichia–Codon language model (Pichia-CLM), a deep learning–based language model for codon optimization to enhance recombinant protein production in the industrially relevant host, *Komagataella phaffii*. Unlike conventional approaches that rely on codon usage bias metrics (CUB)—often providing a global score and ignoring sequence context—Pichia-CLM leverages the host genome to unbiasedly learn the amino acid-to-codon mapping. Prior deep learning models have attempted codon optimization but typically evaluated performance using CUB metrics with limited experimental validation. In contrast, we experimentally validate Pichia-CLM across six diverse protein classes of varying complexity and consistently observe superior expression titers compared to four commercial codon optimization tools. Our findings also reveal the limitations of using CUB metrics, showing a poor correlation between these scores and observed protein yields.

Recombinant proteins are a critical raw material for products across many industries in biotechnology, including biopharmaceuticals, cosmetics, and the food and dairy sectors. Enhancing the production of heterologous protein is essential for cost-effective large-scale manufacturing. Codon optimization–along with other strategies such as cellular engineering, media design, and process optimization–is an important approach to achieve this goal (1, 2).

The need for codon optimization arises from the degeneracy in the genetic code wherein one amino acid can be encoded by more than one codon. There are 61 codons coding for 20 amino acids such that most amino acids (except M and W) can be encoded by at least two and up to six codons. The use of synonymous codons (codons coding for the same amino acid), however, is neither uniform nor random. Across many organisms, certain synonymous codons are often preferred over others, so-called codon usage bias [CUB (3)]. Furthermore, even within a single organism, CUB can vary based on the gene length, gene expression pattern, position, and context of the codon as well as the tRNA abundances (3). Many of these biases can be quantified by several CUB-based metrics such as Codon Adaptation Index [CAI (4)], Codon Pair bias [CPB (5)], Relative codon pair bias [RCPB (6)], tRNA-based indices (7), to highlight a few. The choice of synonymous codons can affect protein production by influencing transcription, mRNA stability, translation, folding, posttranslational modifications (PTMs), and solubility (3, 8, 9). This relationship directly impacts the adaptation or design of coding sequences for expression of heterologous proteins by the host organism.

Several tools and approaches have been developed for codon optimization by considering the CUB of the host. Conventional methods rely on using the most frequent codon in the genome for each amino acid in the sequence (10), based on the observed enrichment of frequent codons in highly expressed genes across different organisms. This approach, however, can lead to translational inefficiencies due to imbalanced tRNA usage and improper folding dynamics (2). More recent approaches use codon sampling methods to select synonymous codons based on their probability of occurrence in the genome (11).

Some alternative methods have formulated the problem as an optimization task, aiming to design sequences that meet certain objectives (10–14). These objectives are often defined through CUB metrics (11, 15), with the most commonly used being the CAI, CPB, RCB, and RCPB. Some approaches also incorporate mRNA stability (12) and rates of protein production as the goals for optimization but these typically require access to appropriate experimental data (14). Despite these advances, such approaches may not reliably produce high-expressing constructs as they fail to capture the position and context-specific complex codon usage patterns observed in natural sequences.

The sole use of CUB metrics ignores the importance of rare codons whose inclusion evolutionarily appears crucial for proper protein folding (16, 17). To address this shortcoming, codon harmonization was developed to design sequences that mimic the natural distribution of codons (18). This restriction, however, limits the approach to only naturally available sequences (19) and overlooks evolutionary drifts and functional selection of codons that might have occurred in the native organism (3, 20).

To address these challenges, we demonstrate here a deep learning–based approach to language modeling to learn the host-specific patterns when mapping amino acid sequences to coding sequences and subsequently use the trained model to design sequences for heterologous protein. Language models offer the advantage of unbiasedly learning the “codon language” directly from the host’s amino acid and coding sequences, accounting not only for the host’s preference but also positional dependencies and long-range context. Recent reports for similar approaches have shown the potential of language models for codon optimization (19, 21–24) by evaluating the performance on theoretical global properties such as CUB metrics [often CAI or its variant (19, 21)], with limited experimental validation for improved production in some cases (22–24).

Here, we developed language models to optimize coding sequences for *Komagataella phaffii* (aka Pichia). This yeast is a widely used host organism in industry due to its high-density cultivations and ability to produce diverse recombinant proteins (25, 26). Moreover, it has the potential to serve as an alternative host to mammalian cell lines for low-cost biomanufacturing of therapeutic proteins and vaccines. Despite their increasing relevance, advanced language model–based tools for codon optimization in *K. phaffii* have been lacking. Here, we show that Pichia–Codon language model (Pichia-CLM) can provide consistent, and often superior, performance relative to commercial algorithms for codon optimization for this host. We show that the embeddings learned for the amino acids and codons grouped them based on physicochemical properties, highlighting the ability of language models to capture physically meaningful trends. We experimentally validated the superior performance of our Pichia-CLM to enhance the production of six proteins from different classes and varied complexities by comparing the performance of Pichia-CLM to commercially available algorithms for codon optimization. We also evaluated the properties of the genetic sequences designed by Pichia-CLM and observed that it avoided negative repeat elements and minimized negative cis-regulatory elements—based on the patterns in the coding sequences, without being explicitly trained on these specific features. Finally, we interpreted the patterns learned by the model by analyzing the properties of the resulting sequences. These results show the potential of language modeling–based approaches to unbiasedly learn patterns and subsequently design robust coding sequences for improved production of recombinant proteins.

## Results

**Fig. 1.**
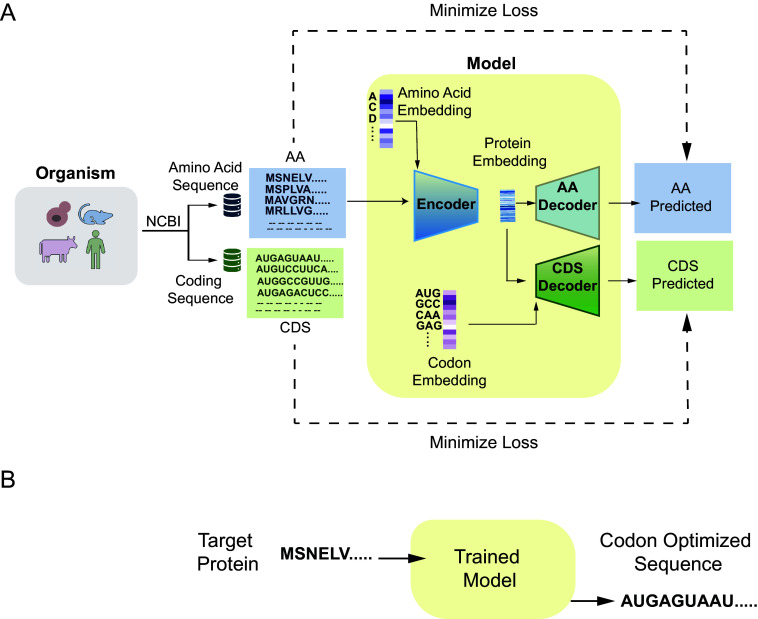
Schematic representation of the workflow and the Pichia–Codon Language Model (Pichia-CLM). (*A*) Workflow during training of the codon language model. (*B*) Application of the codon language model for prediction.

Pichia-CLM uses an encoder–decoder architecture based on Gated Recurrent Unit [GRU (27)], which facilitates the encoding of position and neighborhood context for the selection of codons. It uses the amino acid sequence of the protein as input and generates the corresponding DNA sequence based on the patterns learned from the host’s amino acid and coding sequences (Fig. 1). GRUs provide an improved type of recurrent neural network architecture designed to capture both long and short-term dependencies in sequential data. By using gating mechanisms to regulate the flow of information, GRUs effectively address the vanishing gradient problem commonly found in traditional RNNs. Furthermore, GRUs offer performance comparable to Long Short-Term Memory (LSTM) networks (*SI Appendix*, Table S1), while requiring fewer parameters and lower computational resources, making them a more efficient choice for many sequence modeling tasks. Transformer architectures offer an alternative approach for sequence-to-sequence modeling (28), leveraging an attention mechanism to process entire sequences in parallel. Although Transformers can yield superior performance on large-scale datasets (29, 30), they are computationally and memory intensive. For the species-specific model implemented here, comprising approximately 27,000 sequences (*Materials and Methods*), the Transformer architecture would add unnecessary complexity and computational demands relative to the size of the dataset (30–33). A detailed description of the model hyperparameters, training procedure, and model selection is provided in the *Materials and Methods*.

### Design and Analysis of the Pichia-CLM Architecture.

**Fig. 2.**
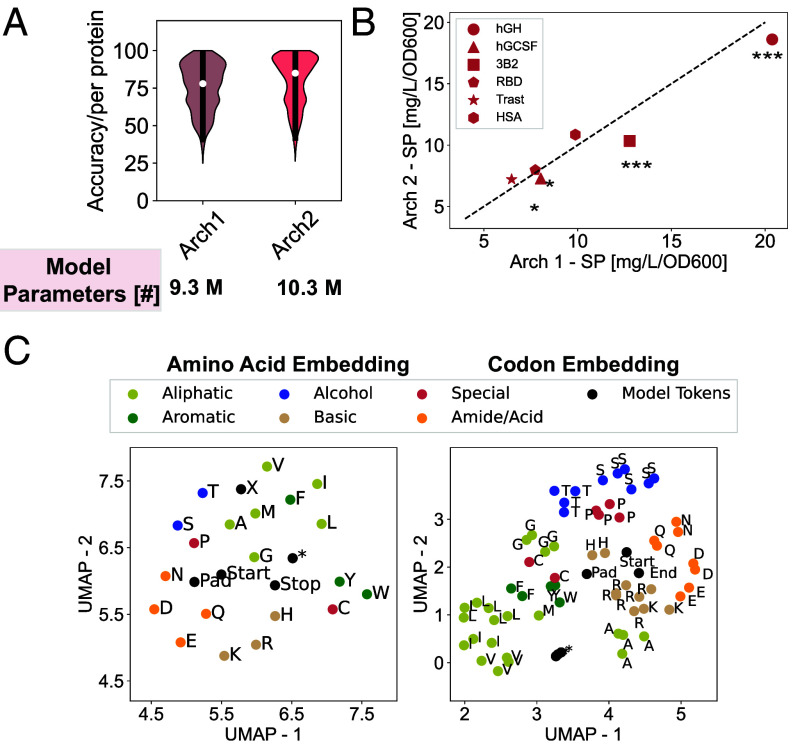
Pichia-CLM architecture determination and inspection. (*A*) Comparison of the predictive accuracy of two alternative architectures using a test set (20% reserved data) (*B*) Scatter plot of the specific productivities of six different recombinant proteins optimized by these models (*C*) UMAP projections of the amino acid and codon embedding learned by the model corresponding to Arch1.

We first sought to assess the performance of our Pichia-CLM approach in silico. We evaluated the accuracy of the model in predicting the coding sequences in a test set (c.f. *Materials and Methods*). This analysis considered two alternative architectures (Arch1 vs Arch2; *SI Appendix*, Table S3) which yielded comparable average validation accuracies during the hyperparameter optimization (*SI Appendix*, Table S4). Both architectures also showed a comparable distribution of prediction accuracy (Fig. 2*A*, *SI Appendix*, Fig. S2) with an average accuracy of 75 to 80% (*SI Appendix*, Table S4) when predicting the coding sequences of proteins in the test set.

We further compared the ability of the two architectures to design sequences for production of heterologous proteins. For this purpose, we evaluated six proteins with varied complexities: human growth hormone (hGH), human growth colony-stimulating factor (hGCSF), a VHH nanobody [3B2 (34)], an engineered variant of the SARS-CoV-2 RBD subunit [RBD (35)], Human Serum Albumin (HSA), and an IgG1 monoclonal antibody trastuzumab (Trast). Both Arch1 and Arch2 resulted in comparable titers and cell-specific productivity (Fig. 2*B*) for all the tested molecules with a marginally, yet statistically significant, improved performance of Arch1 for hGH and 3B2. Arch1 also had fewer parameters than Arch2 with comparable or better predictive capabilities and experimental performance. We, thus, focused on Arch1 for further evaluations.

Next, we inspected the layers of Pichia-CLM to interpret the patterns learned by the model. We analyzed the UMAP projections of the amino acid and the codon embeddings (the numerical representation of these entities) learned by the model. We observed that the algorithm learned embeddings of amino acids that segregated them based on physicochemical properties- Aliphatic, Aromatic, Basic, Acid/Amide, and Alcohols (Fig. 2*C*). The model related hydrophobic amino acids (aliphatic and aromatic) in the UMAP space and similarly clustered the polar amino acids together. Among the polar residues, acid–amide pairs and basic amino acids were also grouped by property. In the embedding space for codons, the model appropriately grouped codons based on their genetic encoding of amino acids. In addition, the grouping observed in the UMAP projection for amino acids was predominantly retained in the UMAP projections of the codon embeddings as well. These results highlight the potential of language models to learn meaningful and physically relevant patterns from sequence data without the need for explicit encoding of such properties.

### Performance of Pichia-CLM for Improved Secretion of Protein in *K. phaffii*.

Following the in-silico evaluation, we then experimentally validated the performance of our approach in generating codon-optimized sequences. First, we evaluated the titers of proteins secreted using gene constructs generated by Pichia-CLM compared to their native coding sequences. We selected three proteins with varied sizes and complexities from humans: hGH, hGCSF, and HSA. We observed that the improvement in yields of secreted protein varied depending on the complexity of the molecule. In general, an improvement of ~25% was observed for proteins such as hGH and hGCSF while an improvement of ~3-fold was observed for HSA. Interestingly, compared to HSA (titer = 45 mg/L), alternative serum albumins such as Bovine Serum Albumin (BSA) and Mouse Serum Albumin (MSA) were secreted more efficiently in *K. phaffii* using the native sequence resulting in titers of 60 mg/L and 100 mg/L, respectively. For these variants of serum albumins, codon optimization resulted in an additional 25% improvement in titers (75 and 135 mg/L).

**Fig. 3.**
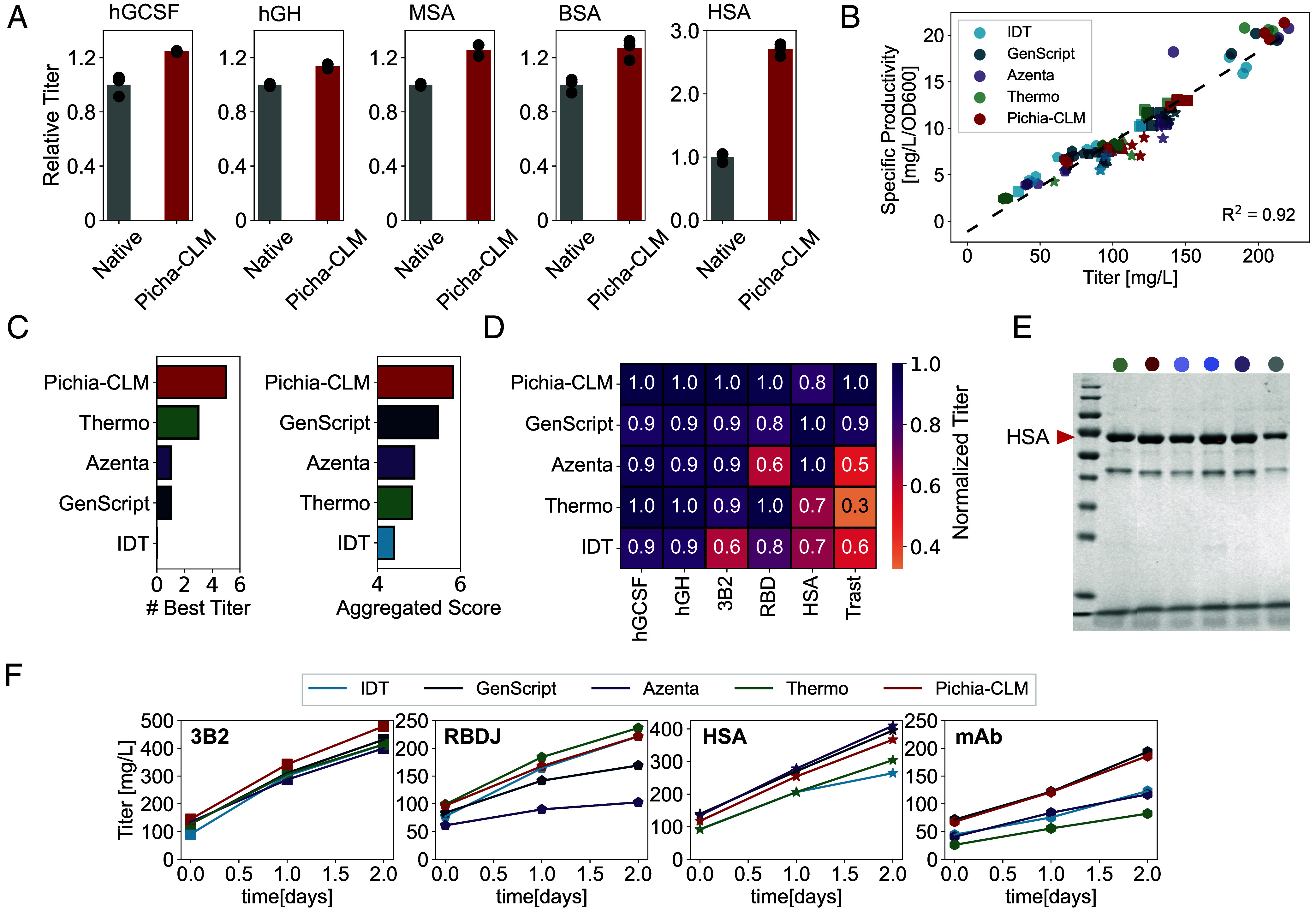
Experimental performance evaluation of Pichia-CLM. (*A*) Bar graphs comparing the secreted protein titers of Pichia-CLM-based codon-optimized variants and the Native coding sequence. (*B*) Scatter plot of cell-specific productivity as a function of titer for different molecules and variants optimized using different algorithms for codon optimization. (*C*) Ranking of the different algorithms for codon optimization based on two metrics: (*i*) number of times an algorithm resulted in the best titer for the test molecules and (*ii*) aggregated score represented by the sum of normalized titers across the molecules tested (normalized to observed molecule-specific maximum titer). (*D*) Molecule-specific comparison of the efficiency of codon optimization using Pichia-CLM and different commercial algorithms. (*E*) SDS-PAGE comparing molecules produced using different codon-optimized sequences for HSA. (*F*) Plots of secreted protein titers as a function of time for four different molecules.

Next, we compared the performance of Pichia-CLM with four commercially available tools for codon optimization: Azenta, IDT, GenScript, and Thermofisher (Thermo). We evaluated the six molecules previously introduced: hGCSF, hGH, 3B2, RBD, HSA, and Trast using two metrics: 1) # BestTiter was used to represent the number of molecules for which a particular approach resulted in the best titer; and 2) Aggregated score, the sum of the relative titer (normalized to maximum value) across different proteins. Overall, we observed that Pichia-CLM outperformed the commercial algorithms (Fig. 3*C*) on both metrics. It resulted in the best titer for five out of six proteins and had a minor reduction (~0.2 units) in the Aggregate score from the reduced protein titers for HSA (Fig. 3*D*). GenScript, on the other hand, yielded the best titer for only one molecule (Trast, Fig. 3*D*), thus ranking low in terms of the # BestTiter. It, however, consistently produced titers within 80 to 100% of the maximum titer, thus emerging as the best candidate among commercial algorithms based on the Aggregated Score.

Thermo ranked second with best titers for three out of six proteins. The Aggregate Score, however, for this approach was ~4.5, resulting from the poor performance of this approach for two out of the three remaining molecules. Interestingly, this approach suffered for proteins with higher complexity, namely HSA and Trast, resulting in 70% and 30% of the maximum titer, respectively. Like GenScript, Azenta also resulted in best titer only for one molecule (HSA, Fig. 3*D*). Unlike GenScript, however, it resulted in titers ranging from as low as 50% and up to 90% of the maximum titer for the remaining molecules. Overall, IDT scored lowest on both metrics. It did not yield the best titer for any of the tested proteins (60 to 90% of the maximum titers, Fig. 3*D*). This observation is consistent with other studies using CHO cells (22). These trends in titers were also preserved in cell-specific productivity (computed as the ratio of titer to optical density) across different proteins and strategies for codon optimization (Fig. 3*B*), as observed by the linear correlation between the two parameters.

Next, we assessed the performance of the Pichia-CLM-designed constructs with respect to product quality since synonymous codon substitution can impact the conformation, stability, and posttranslational modifications of proteins (3, 9). We compared the product and product-related variants generated by our constructs to the ones designed by commercial algorithms and (or) the native sequences using SDS-PAGE. For all the proteins, we observed bands at similar locations for the different codon-optimized constructs and (or) the native sequences (Fig. 3*E* and *SI Appendix*, Fig. S3—gels for other molecules in *SI Appendix*). Finally, to assess whether or not limitations of other factors in cultivation (e.g., nutrient depletion) led to similar maximum titers for sequences optimized with different algorithms, we conducted extended three-day cultivations, with daily additions of 1.5% methanol (v/v) for a subset of molecules of varying complexity (3B2, RBD, HSA, and Trast. For *K. phaffii*, protein expression is induced using methanol (c.f. *Materials and Methods*) to activate AOX1-dependent expression of the transgene. If essential nutrients or other cofactors required for protein synthesis became depleted in the medium, the supplementation with additional methanol would not support production of additional protein, and the measured titers would saturate. We did not observe this behavior in our plate scale studies, and the measured titers increased monotonically over time with addition of methanol.

### Evaluation of the Genetic Sequence Properties.

**Fig. 4.**
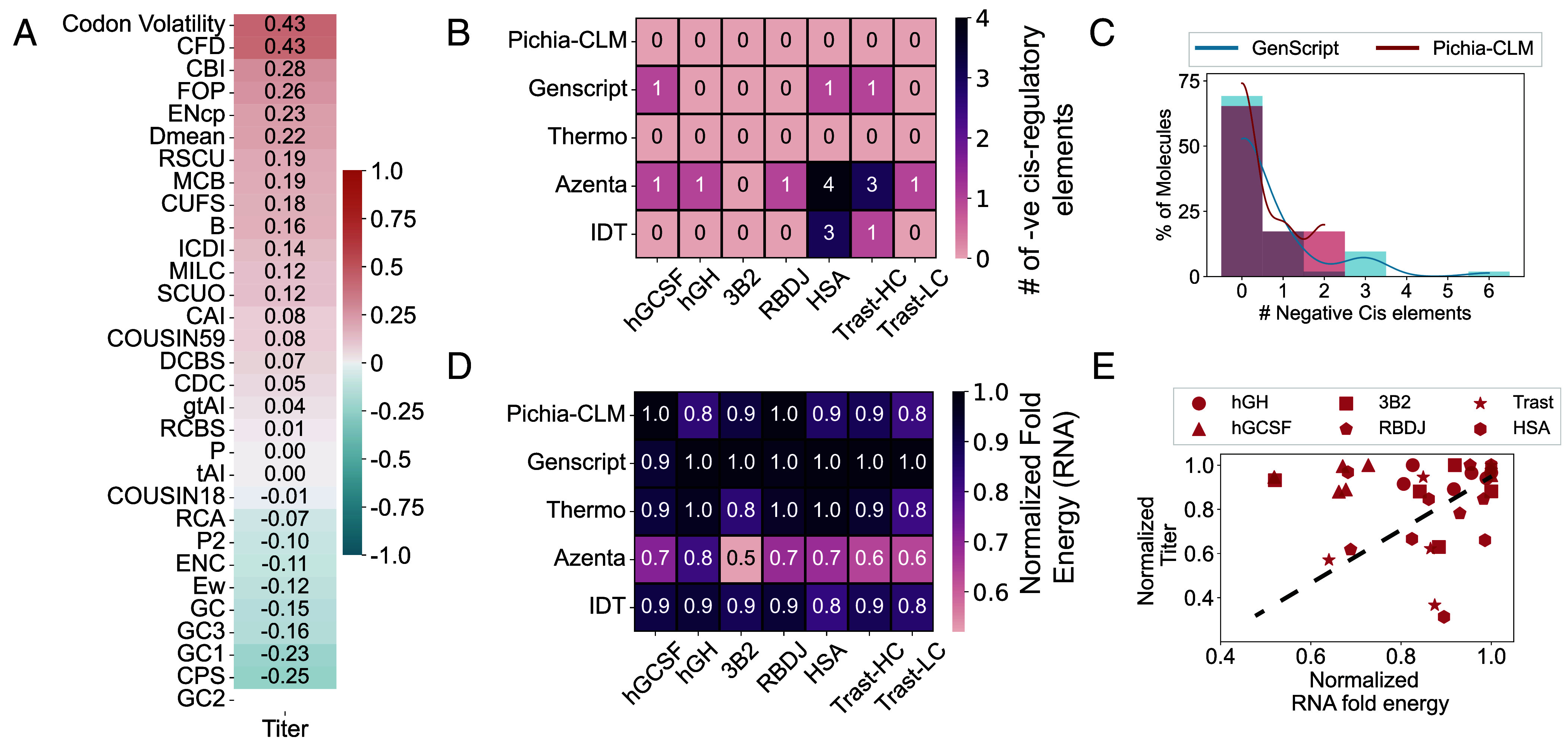
Comparison of Pichia-CLM and commercial algorithms based on calculated/predicted properties of genetic sequence. (*A*) Number of negative cis-regulatory elements predicted in the sequences designed by Pichia-CLM compared to commercial algorithms for the experimentally tested molecules. (*B*) Comparison of the distribution of negative cis-regulatory elements predicted for 52 biotechnologically relevant benchmark proteins optimized using Pichia-CLM and GenScript. (*C*) Comparison between Pichia-CLM and commercial algorithms for RNA stability based on free energy of predicted RNA structures for the different constructs. (*D*) Relationship between RNA stability and observed titers for the experimentally tested constructs. (*E*) Correlation between titer and the different codon bias indices available.

After validating the performance of our Pichia-CLM approach for the production of heterologous proteins, we then investigated the properties of the genetic sequence for the different designed constructs. Codon optimization, including that enabled by other reported protein language models, is typically performed or evaluated based on one of many CUB metrics (19, 21–24). So, we sought to assess the correlation between these CUB metrics (c.f. *Materials and Methods*) and production using the data collected for the six tested proteins. We observed that none of these metrics showed a consistent, high correlation with the titers across the different proteins. For instance, in the case of HSA (Fig. 4*A*), a maximum positive correlation of only 0.43 was observed with codon volatility and codon frequency distribution (CFD), while maximum negative correlation of only 0.25 was observed with codon pair score (CPS). For RBDJ (*SI Appendix*, Fig. S4), on the other hand, a high positive correlation (~ 0.9) was observed with GC3 and GC content with a negative correlation of 0.39 with CAI. It is interesting to observe that CAI—the typical objective that is maximized in codon optimization, negatively correlated with titers. CAI was also observed to show high negative correlation of 0.81 with titer for Trast (*SI Appendix*, Fig. S4). In contrast to HSA, where CPS showed a minor negative correlation with titer, CPS was highly positively correlated with titer for Trast (coefficient = 0.9, *SI Appendix*, Fig. S4).

These varied patterns in correlation between CUB metrics and titers underscore the limitations of global metrics computed over an entire sequence to effectively represent features relevant for production of heterologous proteins. Furthermore, it highlights the need for alternative metrics to evaluate tools for codon optimization, potentially supported by rigorous experimental validation across diverse proteins. Several combinations of codons may result in the same CUB metric, but the local positioning and the context of the different codons can play a crucial role in determining the efficiency of expression of a certain construct, as demonstrated by the Pichia-CLM model here.

We next evaluated the presence of negative cis-regulatory elements in the different codon-optimized constructs. These elements can interfere with the host’s regulatory mechanisms (19, 21) and, thus, it may be optimal to avoid them in heterologous DNA sequences. For the six proteins tested, the constructs designed using the Pichia-CLM approach resulted in no negative cis-regulatory elements (Fig. 4*B*). Unsurprisingly, Thermo’s constructs also did not possess any negative cis-regulatory element, as this feature is explicitly programmed into their codon optimization pipeline (10). Sequences from GenScript had one negative cis-regulatory element in three out of the six tested proteins while both Azenta and IDT resulted in sequences with 3 to 4 negative cis-regulatory elements for at least one of the tested proteins (Fig. 4*B*). We further analyzed the presence of negative cis-regulatory elements in the constructs designed by Pichia-CLM for 52 biotechnologically relevant proteins, recently summarized in (19). For 75% of these proteins, the Pichia-CLM approach resulted in no negative cis-regulatory elements, with the remaining 25% of the proteins having at most two elements (Fig. 4*C*). By comparison, GenScript—the best performing commercial algorithm in protein production generated constructs with three to six elements for 15% of these proteins (Fig. 4*C*). Additionally, all the constructs designed by Pichia-CLM, including the ones designed for the 52 biotechnologically relevant proteins, were devoid of negative repeat elements (SupplementalData, RCA.xlsx), similar to the commercial algorithms. Though these characteristics of the genetic sequences were not explicitly enforced in the language model, it could learn these features based on the patterns observed in the data, highlighting the potential of this approach to identify regulatory features encoded in a host’s genome.

Finally, we assessed the predicted mRNA stability of the different constructs by computing the minimum folding energy of the predicted RNA secondary structure. Substitutions of synonymous codons can affect mRNA secondary structure and subsequently the stability (8, 16). mRNA stability, in turn, has been shown to positively impact protein translation by improving the interaction with RNA-binding proteins (3). In addition, it also increases the half-life of the mRNAs, thus increasing the time an mRNA is available for translation. For the six experimentally tested proteins, the Pichia-CLM-designed constructs yielded 80 to 100% of the minimum folding energy observed among all the constructs designed for each molecule (Fig. 4*D*). Interestingly, despite the differences in observed protein production, all the commercial algorithms except Azenta also resulted in stable RNA secondary structures (Fig. 4*D*). Though we observed a general increase in titer with higher RNA stability, we also identified constructs with lower stabilities resulting in higher titers and vice-versa (Fig. 4*E*). It is noteworthy that for smaller proteins such as hGCSF and 3B2, higher titers were achieved despite lower RNA stability while for complex proteins such as HSA and Trast, higher mRNA stability was not the only determining factor for increasing production of protein (Fig. 4*E*). Subsequently, no strong correlations can be inferred between titer and mRNA stability, reiterating the complexity and combinatorial impact of various factors on protein production. Taken together, these observations suggest that Pichia-CLM not only results in high protein-producing constructs but is also capable of learning relevant genetic sequence features, balancing complex interaction of these features and subsequently designing robust sequences for expression in the host.

### Interpretation of Codon Optimization Principles of the Different Algorithms.

**Fig. 5.**
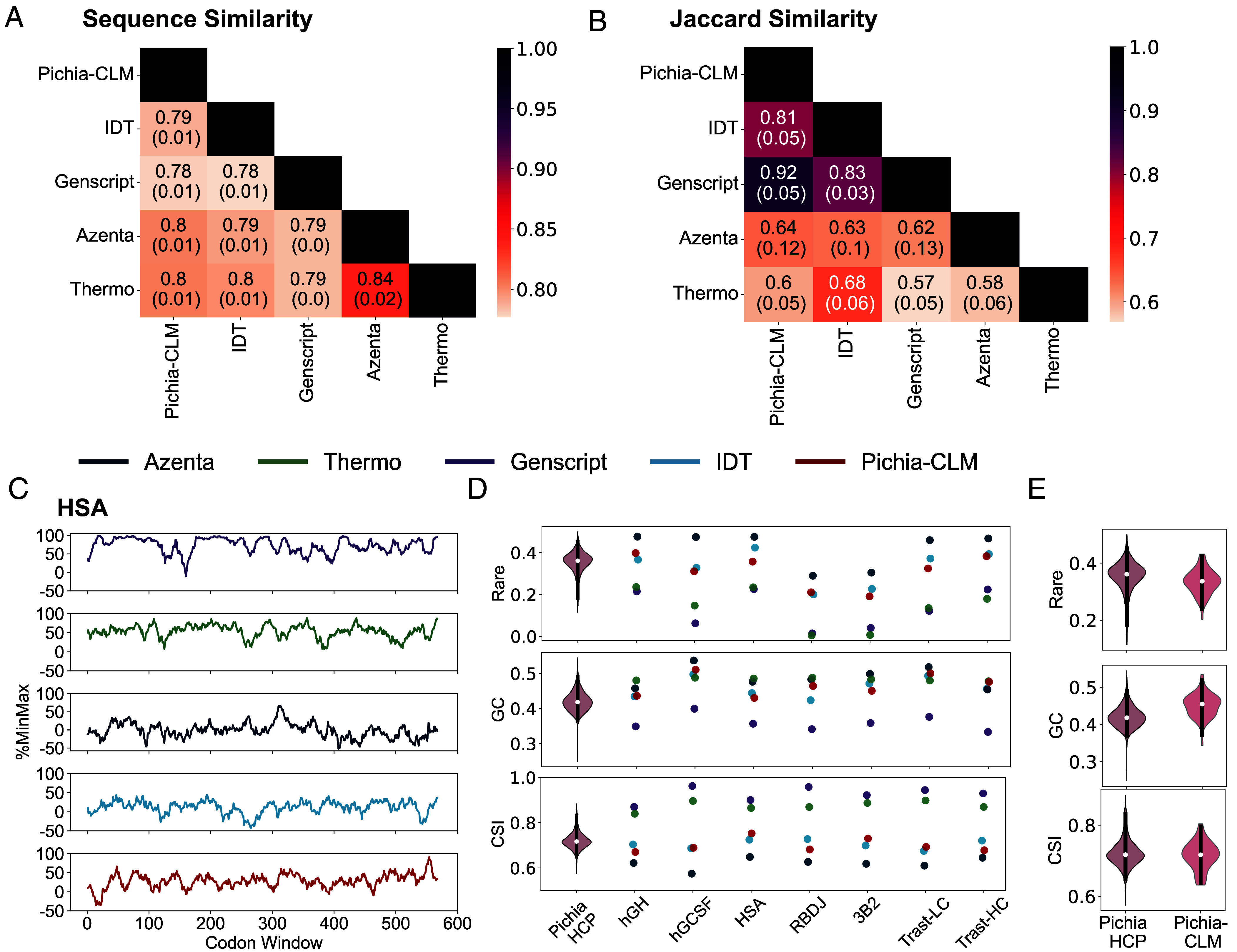
Characteristics of the sequences optimized by Pichia-CLM and commercial algorithms. (*A*) Heatmap of the sequence similarity among the sequences generated by different algorithms reported as the average and SD across different molecules. (*B*) Heatmap of Jaccard similarity among the sequences generated by different algorithms reported as the average and SD across different molecules. (*C*) %Min-Max plots of the different codon-optimized sequences for HSA. (*D*) Rare codon content, GC content, and CSI of the codon-optimized sequences generated by various algorithms for the six different proteins, compared against the distribution of these properties computed for Pichia’s host cell proteins. (*E*) Distribution of the Rare codon content, GC content, and CSI of the Pichia-CLM generated constructs for 52 biotechnologically relevant proteins compared against the distribution of these properties for Pichia’s host cell proteins.

These variations in production and characteristics of the genetic sequences among the different algorithms are a result of the differing principles each uses for codon optimization. As a result of these underlying differences, there is only 80% sequence similarity between the constructs designed by the Pichia-CLM and the various commercial tools (Fig. 5*A*). Among the different commercial algorithms, Azenta and Thermo shared a slightly higher sequence similarity of 84% with each other.

Interestingly, however, Pichia-CLM showed 81% and 92% Jaccard similarity with IDT and GenScript, respectively (Fig. 5*B*). Sequence similarity is a position-specific metric that quantifies the number of positions that uses the same codon between two constructs. Jaccard similarity metrics, on the other hand, is not position-specific and measures the proportion of the codons shared between two constructs relative to the total codons used across both the constructs. A higher similarity on the Jaccard scoring between Pichia-CLM, GenScript, and IDT, thus, indicates that a similar set of codons is used by these algorithms. In contrast, Pichia-CLM shared only ~60% Jaccard similarity with Azenta and Thermo constructs indicating the use of significantly different sets of codons in their designs.

The differences in choices of codons was further obvious when we compared the %MinMax profiles (36) of the different constructs (Fig. 5*C* and *SI Appendix*, Fig. S5). The %MinMax profiles of Azenta and Thermo had positive scores in all the windows, indicating that these tools relied predominantly on the use of the frequent codons. This bin is also reflected in the lower percentage of rare codons (Fig. 5*D*, %Rare) and a higher CSI (Fig. 5*D*, %CSI) for these algorithms. This result was unsurprising since the strategies for codon optimization used by Azenta rely on replacement with frequent codon (37) while Thermo’s algorithm samples based on frequency distribution of codons to maximize CAI (10). These two approaches, however, differ in the GC content (Fig. 5*D*, %GC), with Thermo’s constructs presenting higher GC content compared to Azenta. This outcome is a result of Thermo’s optimization strategy that includes GC content as one of the factors in their quality scores for selecting the codon at each position (10).

Unlike Azenta and Thermo, Pichia-CLM, GenScript, and IDT all use both frequent and rare codons in their constructs, maintaining a %MinMax scores between −50% and +50% (Fig. 5*C* and *SI Appendix*, Fig. S5), thus explaining the higher Jaccard similarity among them. These three approaches also gave similar rare codon and GC content (Fig. 5*D*). Despite these similarities, there are, however, appreciable differences in protein production achieved from these algorithms. The experimental performance of the constructs generated by Pichia-CLM and IDT varied significantly despite the similar rare codon content, GC content, and CSI. Pichia-CLM ranked the highest while IDT was ranked the lowest across both the metrics evaluated (Fig. 3*C*). These differences can be attributed to the variations in the %MinMax profiles (Fig. 5*C*) and the deviations in sequence similarities of the constructs (Fig. 5*A*), thus underscoring the importance of considering codon position and contextual effects in codon optimization, rather than relying solely on global metrics. Furthermore, contrary to the prevailing principles of codon optimization—which often aims to maximize the Codon Similarity Index (CSI)—our experimental data reveal that constructs with lower CSI values can, in fact, lead to higher protein expression. Notably, the constructs generated by the two best-performing algorithms (Pichia-CLM and GenScript) exhibited the lowest CSI among all tested designs yet yielded superior protein production. This finding further reiterates the observations made earlier regarding negative correlation of CAI with titer observed for Trast and RBDJ (*SI Appendix*, Fig. S4), highlighting the limits of applying CUB metrics for codon optimization.

Interestingly, the sequences designed by Pichia-CLM exhibit rare codon usage, GC content, and CSI that fall within the 25 to 75 percentile distribution of the corresponding properties observed for *K. phaffii*’s host cell proteins (HCPs). To further validate this trend, we inspected these properties for the Pichia-CLM codon-optimized constructs of 52 biotechnologically relevant proteins (19). The distribution of the properties for the 52 heterologous proteins aligned well with the distribution of Pichia’s HCPs with both the distributions having comparable median values. Overall, this behavior indicates that the Pichia-CLM has learned *K. phaffii*’s language of mapping amino acid sequences to coding sequences and, as a result, generates sequences similar in genetic sequence properties to that of the intrinsic HCPs.

## Discussion

In this study, we present a language model–based pipeline for codon optimization for an industrially relevant host organism *K. phaffii*. We validated the performance of Pichia-CLM on both experimental and theoretical properties. First, we demonstrated that our approach consistently outperformed commercial tools for codon optimization to improve the production of multiple proteins with varied complexities. In addition, we also demonstrated its ability to generate constructs with favorable properties for the genetic sequence such as avoiding negative repeat elements, minimizing negative cis-regulatory elements, and stable mRNA secondary structures, without being explicitly trained to do so. We further showed that the model appropriately learned the codon families of the amino acids. Furthermore, it also learned similarities in physicochemical properties among amino acids directly from the sequence data, thus highlighting the ability of these types of approaches to learn physically relevant behavior. Finally, we demonstrated that the Pichia-CLM generates constructs with sequence properties similar to *K. phaffii*’s host cell proteins, likely resulting from the model applying the language learned from the host sequence data to the heterologous proteins.

The selection of synonymous codons varies across different species, among different proteins and within the protein depending upon the position and context. As a result, it is important to suitably adapt coding sequences of heterologous proteins, through codon optimization, to enhance compatibility and subsequent protein production. The state-of-the-art approaches for codon optimization, however, rely on the use of biased metrics defined to quantify CUB based on various hypotheses. As we show here, these CUB metrics do not consistently correlate with protein production for different proteins, highlighting the inability of one global metric to capture the principles of synonymous codon selection effectively.

The language modeling–based approach demonstrated here presents an unbiased learning paradigm of the principles connecting amino acid sequences and coding sequences in the host organism. Furthermore, unlike the state-of-the-art approaches, this approach can encode positional and contextual information in its learning. While such approaches have been demonstrated previously, the experimental validations of the proposed models have been limited. In this study, we validated our Pichia-CLM pipeline experimentally on six distinct proteins with varied complexities as well as the theoretical properties using 52 additional biotechnologically relevant proteins. Furthermore, we here demonstrated the ability of this codon optimization to enhance production of secreted heterologous proteins. The tool could, however, also be used to enhance production of *K. phaffii*’s own native proteins or of other genes integrated into the host (e.g., chaperones; glycosyltransferases).

One potential limitation of this study is that the model was trained for a single host organism (*K. phaffii)*. The model could, however, be adapted for other species by training on their respective genomes such as *Homo sapiens*, *Mus musculus,* and *Bos taurus* (*SI Appendix*, Table S5). By including species-specific start tokens, this model can also be extended to incorporate multiple species, enabling the learning of general and species-specific patterns in codon usage and associated organisms based on similarities in synonymous codon preferences (*SI Appendix*, Fig. S6). Increasing the number of species in a multispecies model and expanding the included datasets could also benefit from more advanced architectures like transformer-based models than the GRU-based approach here.

## Materials and Methods

### Dataset Collection and Preparation.

To train the Pichia-CLM, amino acid and coding sequence data for two variants of *Pichia Pastoris*: CBS7435 (commonly referred to as *K*. *phaffii*) and GS115 were collected from NCBI. These data were supplemented by the genome characterized and annotated previously in our lab (38) for GS115, *K*. *phaffii* (NRRL Y11430) and *K*. *pastoris*. A total of ~27,000 amino acid–coding sequence pairs were used. The amino acids and codons were tokenized with the addition of <START> and <END> tokens to mark the beginning and end of the sequences. <PAD> tokens were used to match the length of different sequences in the data to facilitate batch processing of the data by the model. 20% of the dataset was randomly generated and reserved as the test set to evaluate model predictive performance in-silico.

### Architecture Setup and the Hyperparameter Optimization.

Encoder–decoder architecture was adapted to build the codon language model using Gated Recurrent Units capable of encoding position information and long-term memories. The Encoder network sequentially learns a numerical representation of the amino acid sequences which is then supplied to the Decoder network to generate the coding sequence. We used a bidirectional GRU in the encoder network to capture context information on either side of the current encoding position. In the decoder, however, we used a GRU to create a numerical representation of a given codon position. The decoder GRU network is followed by a fully connected network that takes the numerical vector generated by the decoder GRU and predicts the probability of the different codons. This configuration resulted in the following hyperparameters: i) dimension of the amino acid embedding, ii) dimension of the codon embedding, iii) units in the encoder layers, iv) size of the decoder dense layer for codons, and v) size of the decoder dense layer of amino acid. The ranges of the different hyperparameters tested in the hyperparameter optimization are detailed in *SI Appendix*, Table S2.

The dataset was subsequently split into 80% training and 20% test set reserved to evaluate the predictive capabilities of the model. Early stop for parameter optimization was implemented through validation set (20% of the training set). In addition, the minimization of validation loss (sparse categorical crossentropy loss function) was also used as the objective for hyperparameter optimization to select the best architecture. Hyperparameter optimization was implemented using a global optimization strategy called Bayesian optimization (39) using the in-house codes developed for (40). The different combinations of hyperparameter values tested during the hyperparameter optimization routine and the corresponding accuracies are summarized in *SI Appendix*, Fig. S1.

During training of the model, the original coding sequences with the actual codons were used as input to the decoder layers. When using the model for prediction, however, the predicted codon at the previous position is used as the input for the next position, thus making the model fully predictive. The sequential prediction is halted when a stop codon is encountered. The validation errors and test errors of the model are summarized in *SI Appendix*, Table S3 along with the final selected hyperparameters.

Once the predictive capability of the selected architecture is evaluated on the reserved test set, a final model is trained with the complete dataset, while still using early stop during the parameter optimization to avoid overfitting. This model trained on the complete dataset is used for designing the coding sequences for the heterologous proteins.

### Genetic Sequence Properties.

GenScript’s RCA tool (41) (https://www.genscript.com/tools/rare-codon-analysis) was used to compute the negative cis-regulatory elements, negative repeat elements and the various CUB evaluated in this work. The minimum folding energy computed based on the predicted RNA secondary structure was obtained using the RNAStructure (https://rna.urmc.rochester.edu/RNAstructureWeb/Servers/Fold/Fold.html).

The following properties were computed for every coding sequence in the host’s genome as well as for the test proteins.

1. Relative adaptiveness
  $$w_{\mathrm{ij}}=\frac{x_{\mathrm{ij}}}{x_{i,max}},$$
  where $x_{\mathrm{ij}}$ is the frequency of codon j of amino acid i, and $x_{i,max}$ is the frequency of the most used codon.
2. CSI
  $$CSI=\exp\left(\frac{1}{L}\sum_{k=1}^{L}\ln\left(w_{k}\right)\right),$$
  where L is the length of the DNA sequence, and $w_{k}$ is the relative adaptiveness
3. GC
  $$GC=\frac{\left(G+C\right)}{L},$$
  where G and C represent the respective number of these nucleotide bases in the DNA sequence, and L represents the length of the DNA sequence
4. ORare fraction
  $${\mathrm{Id}}_{k}=\left\{\begin{array}{cc} 1, & x_{\mathrm{ij}}<0.3,x_{\mathrm{ij}}<{\mathrm{Avg}}_{i}\\ 0, & x_{\mathrm{ij}}\geq 0.3\;x_{\mathrm{ij}}>{\mathrm{Avg}}_{i} \end{array}\right.,$$
  $$Rare=\sum_{k=1}^{L}{\mathrm{Id}}_{k},$$
  where $x_{\mathrm{ij}}$ is the frequency of codon j of amino acid i and ${\mathrm{Avg}}_{i}$ is defined as the probability of picking a codon for an amino acid i assuming a uniform probability to pick any codon.
5. Sequence similarity
  $$SeqSimilarity\left(X,Y\right)=\frac{1}{L}\sum_{i=1}^{L}\delta (x_{i},y_{i}),$$
  where X and Y are two different DNA sequences, L is the length of the DNA sequence, and δ is the Kronecker delta function.
6. Jaccard similarity
  $$J\left(X,Y\right)=\frac{A\cap B}{A\cup B},$$
  where X and Y are two different DNA sequences and A and B indicate their respective codon sets.

### Cloning Procedure.

The various codon-optimized constructs for the different proteins were obtained as DNA fragments (TWIST Biosciences) and assembled into custom vector using HiFi assembly (New England Biolabs, Cat #E2621). The plasmid backbone was kept identical for the different constructs. The strains were obtained by transforming the respective linearized plasmids into the modified base strain [AltHost Research Consortium Strain S-63 (RCR2_D196E, RVB1_K8E) (38)] using transformation protocol previously described in (42). For all constructs evaluated, linearized plasmids encoding the heterologous genes were integrated via roll-in integration at the AOX1 locus in the genome, and expression modulated by induction with methanol. The different codon optimization tools employ their own distinct principles, and the optimal copy number for each may differ among optimized constructs. Since the general goal when expressing heterologous proteins is to maximize production regardless of other factors, we chose to compare the performance of the codon-optimized sequences under typical conditions pragmatically used in the lab—that is, identifying the most productive clones to compare. This approach normalizes the potential interaction between copy number and codon optimization selected and allows a head-to-head comparison of the best performing clones for each. For all proteins, except trastuzumab, *Saccharomyces cerevisiae* α-mating factor was used as the signal peptide to enable secretion of the recombinant proteins. For trastuzumab, preregion of *S. cerevisiae* OST1 and proregion of α-mating factor was used to generate the signal peptide. Transformed *K*. *phaffii* cells were plated on solid YPD agarose plates with Zeocin antibiotic resistance. Eight clones were selected and cultivated using the cultivation protocol (c.f. 4.2.2). Expression was compared through SDS-PAGE analysis and the clone exhibiting highest expression was chosen. SDS-PAGE was carried out to confirm protein bands of the right size and inspect product-related variants. Analysis was performed under reducing condition using Invitrogen NovexTM 12% Tris-Glycine Plus Midi Gel (Cat# WXP01226BOX) following the manufacturer recommended protocol. Gels were stained with ReadyBlue™ (Sigma-Aldrich Cat#RSB1-1L).

### *K*. *phaffii* Cultivation and Analytics.

Cultivation experiments to compare the strains bearing different codon-optimized constructs were performed in Axygen twenty-four deep well square plates (total volume 10 mL, working volume of 3 mL) at room temperature on Benchmark Orbi-Shaker^TM^ plate shakers (600 rpm). Complex media, the so-called BMxY—1.34% nitrogen base w/o amino acids (Difco Yeast Nitrogen Base w/o Amino Acids, Cat# 291920), 1% yeast extract (DifcoTM Yeast Extract, Cat# 210929), 2% peptone (BactoTM Peptone, Cat# 211677), potassium phosphate buffer at pH 6.5 was used as the basal media for the cultivation. Outgrowth for biomass accumulation was facilitated using 4% glycerol (Sigma-Aldrich, Cat#G7893) as the carbon source and recombinant protein production was induced using 1.5% methanol (Sigma-Aldrich, Cat#179337). Cells were streaked from the master cell bank of the various strains on YPD plates with Zeocin antibiotic resistance, allowed to grow in 30 °C incubators (Eppendorf, New Brunswick Innova® 42) for 48 h. Overnight cultures in YPD were started by scooping cells using a 1 uL loop. Cultivations were inoculated at 0.1 OD600 from these overnight cultures, grown for 24 h, pelleted, and resuspended in fresh media containing methanol to induce recombinant gene expression. Supernatant samples were collected after 24 h of production, filtered, and analyzed using High-Performance Liquid Chromatography (HPLC; Agilent Infinity 1260) to quantify titer. For all the molecules, except trastuzumab, reverse-phase chromatography (Agilent Technologies, Cat# PL1612-1801) while trastuzumab titers were quantified using affinity chromatography using Biomonolith protein A column (Agilent Technologies, Cat#5190-6903). 1.5% methanol was spiked every 24 h when studying cultivation performance for extended durations. Biological triplicates were run for all cultivation experiments.

## Supplementary Material

Appendix 01 (PDF)

Dataset S01 (XLSX)

## Data Availability

Model parameters, Codes for analysis, data have been deposited in GitHub https://github.com/NHarini-1995/PichiaCLM.git (43). Study data are included in the article and/or supporting information.
