# Supplementary material for: Pichia-CLM: A language model–based codon optimization pipeline for Komagataella phaffii
Source: Proc Natl Acad Sci U S A. 2026 Feb 17;123(8):e2522052123. doi: 10.1073/pnas.2522052123 (PMC12933070; doi:10.1073/pnas.2522052123)
Supplement: Supplementary file 1 — Appendix 01 (PDF) [file pnas.2522052123.sapp.pdf]

Supplementary Information

1. LSTM vs GRU

The LSTM model was trained like the GRU model with the same hyperparameter optimization routine and the same boundaries (Table S2).

Table S1: Model performance of the best GRU vs LSTM architectures

**Assessment Parameter**	**Arch 1**	**Arch 2**	**LSTM**
Validation Accuracy [%]	87.0	86.0	87.0
Test Accuracy [%]	75.0	79.0	
Model Parameter [#]	9.3 M	10.3 M	13.7M

2. Hyperparameter optimization

Table S2: Ranges of the different hyperparameters used for the hyperparameter optimization of the Pichia-CLM.

**Parameters**	**Min**	**Max**
Embedding Size – Amino Acid	30	260
Embedding Size – Codons	30	260
Encoder Size	60	515
Hidden Layer Size – Amino Acid decoder	30	260
Hidden Layer Size – CDS decoder	30	260
Dropout – Codons	0	0.9
Dropout – Amino Acid	0	0.9

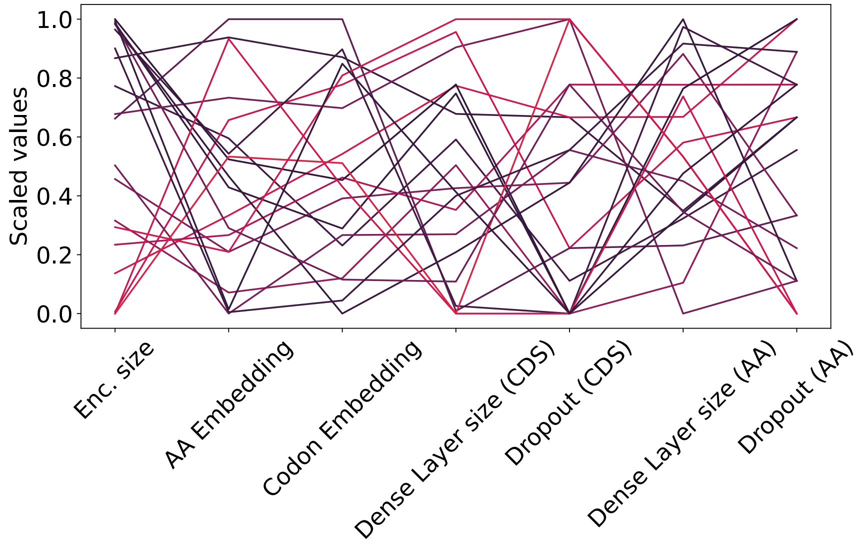

Fig S1: Parallel coordinate plot showing the combinations of different hyperparameters tested during hyperparameter optimization with the different lines colored by the validation accuracy of the respective models such that higher accuracy is represented with a darker shade.

3. Pichia Codon Language Model Architecture Parameters

Table S3: Optimized hyperparameters for the two final competing deep learning model trained on *K.phaffii's* host protein and coding sequence data.

**Parameters**	**Arch 1**	**Arch 2**
Embedding Size – Amino Acid	42	149
Embedding Size – Codons	224	135
Encoder Size	510	513
Hidden Layer Size – Amino Acid decoder	139	205
Hidden Layer Size – CDS decoder	125	209
Dropout – Codons	0.0	0.0
Dropout – Amino Acid	0.7	0.9

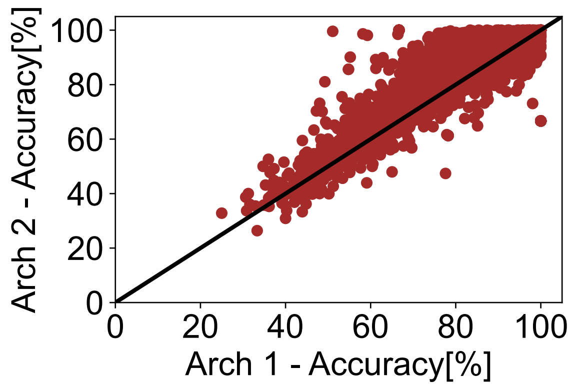

Fig S2: Scatter plot demonstrating the agreement of predictive accuracy of both the architectures of Pichia Language models (Arch1 and Arch 2)

4. Model Performance of the Language Model

Table S4: Model performance of the different codon optimization models

Assessment Parameter	Arch 1	Arch 2
Validation Accuracy [%]	87.0	86.0
Test Accuracy [%]	75.0	79.0
Model Parameter [#]	9.3 M	10.3 M

5. Comparison of product quality between different codon optimized constructs for various molecules

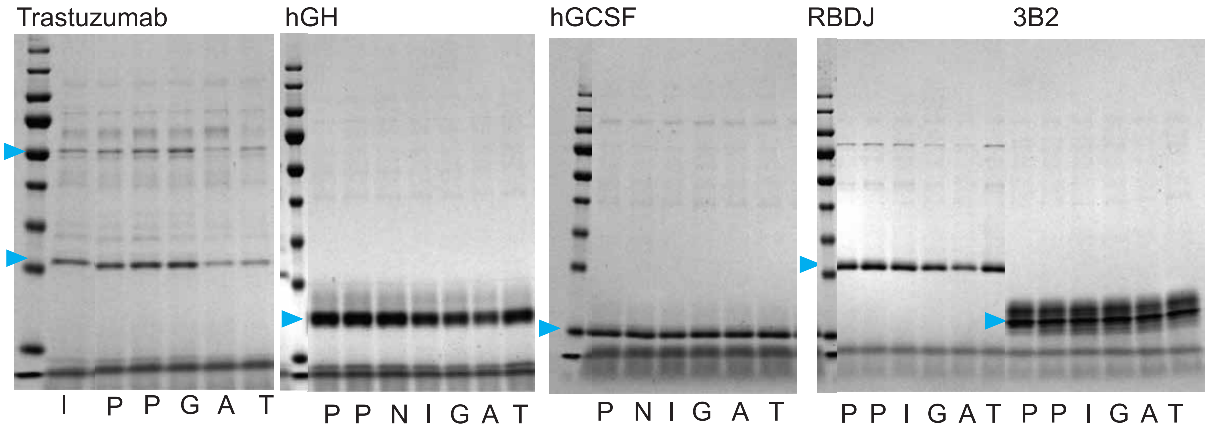

Fig S3: SDS-PAGE gel images comparing the codon optimized constructs for five tested proteins.

6. Correlation between Codon usage bias metrics and titer for the different molecules

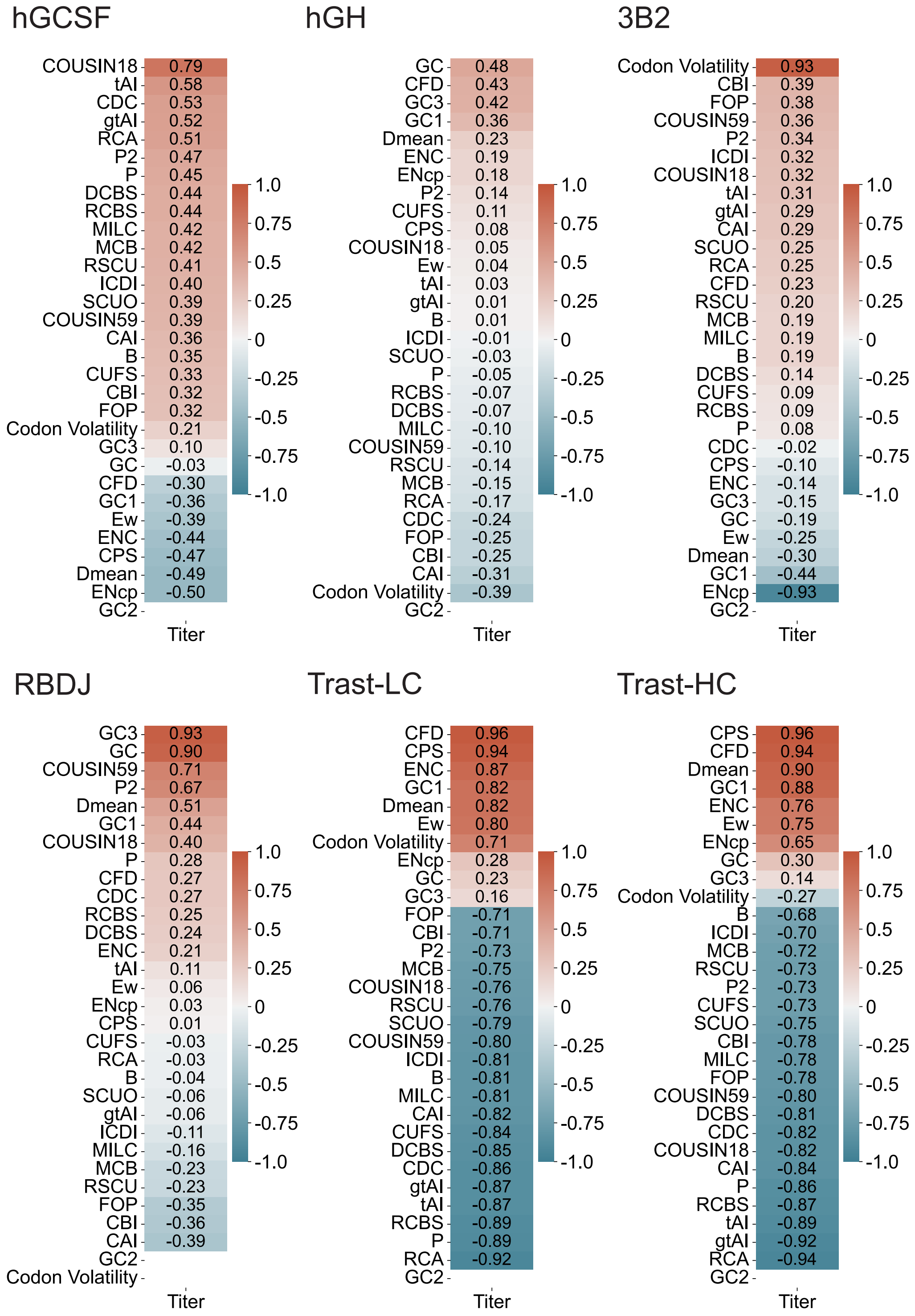

Fig S4: Correlation of the different codon usage bias metrics to titer for five tested proteins.

7. %MinMax profiles for the different molecules

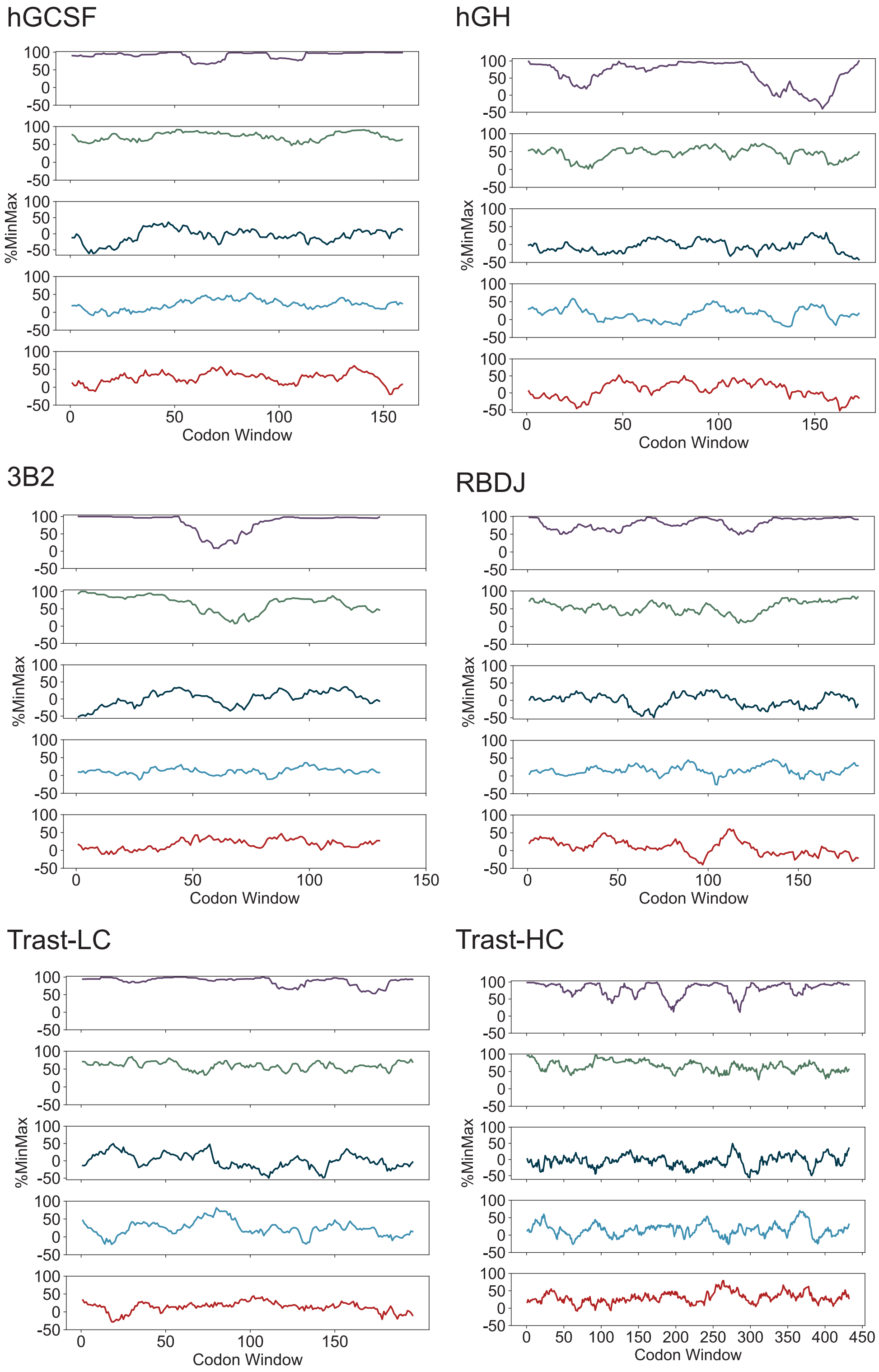

Fig S5: %MinMax profiles for the different codon optimized constructs for five tested proteins.

8. Species-specific Model performance for other host organisms

The best architecture identified for Pichia-CLM was directly applied for three other host organisms: *Mus musculus, Bos taurus and Homo sapiens.*

Table S5: Model performance of the different species-specific codon optimization models

Organism	Validation Accuracy [%]
K.phaffii	87.0
Mus musculus	85.2
Bos taurus	82.0
Homo sapiens	86.4

9. Multi-species model considering six species

The encoder- decoder architecture was adapted to include multiple species differentiated in the model using an organism specific start tokens. As a proof-of-concept, six species- *K. phaffii*, *E. coli*, *S. Cerevisiae*, *Mus musculus* (Mouse), Chinese Hamster Ovary cells and *Homo sapiens* (Human) -were used to develop the multi-species model. To address the increased complexity, the hyperparameter bounds were expanded.

Table S6: Ranges of the different hyperparameters used for the hyperparameter optimization and the optimized hyperparameter with the corresponding validation accuracy.

Parameters	Min	Max	Optimized
Embedding Size – Amino Acid	30	515	86
Embedding Size – Codons	30	515	33
Encoder Size	60	2000	1898
Hidden Layer Size – Amino Acid decoder	30	515	318
Hidden Layer Size – CDS decoder	30	515	513
Dropout – Codons	0	0.9	0.2
Dropout – Amino Acid	0	0.9	0.7
Validation Accuracy [%]			87.8

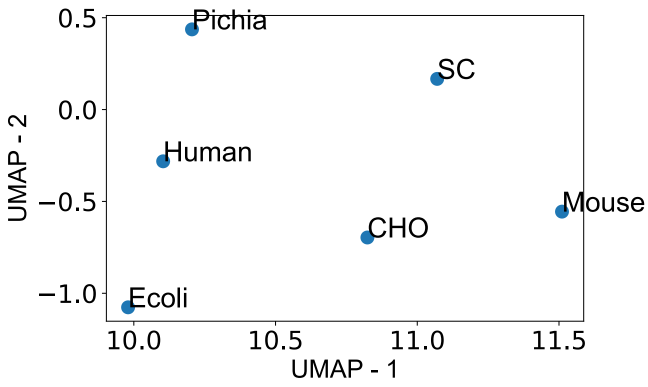

Fig S6: UMAP projection of the embedding learned for the species-specific start tokens in the multi-species codon optimization model.
